# A Tissue-Chopping Based Immunofluorescence Staining Method for Chloroplast Proteins

**DOI:** 10.3389/fpls.2022.910569

**Published:** 2022-05-19

**Authors:** Lulu Wang, Mingdong Tang, Wenwen Huang, Jinjie An, Xiaomin Liu, Hongbo Gao

**Affiliations:** ^1^National Engineering Research Center of Tree Breeding and Ecological Restoration, Beijing Forestry University, Beijing, China; ^2^College of Biological Sciences and Technology, Beijing Forestry University, Beijing, China

**Keywords:** tissue-chopping, immunofluorescence staining, tissue lysis, chloroplast, protein localization

## Abstract

Immunofluorescence staining is an important method for detecting the localization of proteins in the cell. It is also frequently used in the localization study of chloroplast-division proteins. Although this method has been improved before by using protoplasts, it still has some limitations. Now we developed a new method to make it much easier. We just broke the plant leaf tissue with a serrated blade, stained the samples directly, and simply lysed the tissue into separatable cells. The localization of the target protein can then be observed with a clear view. Since this method directly uses broken leaf pieces, it is very fast. It can also be applied to the plants in which protoplasts are difficult to prepare. We first used this method to observe the localization of a chloroplast division protein FtsZ1 in the wild-type Arabidopsis. A ring was clearly seen in the middle of chloroplasts. In addition, we used this method to analyze the localization of FtsZ1 in *arc3* and *pdv2* mutants, as well as in dozens of other species, including some woody plants. This new immunofluorescence staining method is not only easy to use, but also has a wide applicability in various plants.

## Introduction

Chloroplasts are photosynthetic organelles encapsuled by double membranes. They propagate by binary division. Chloroplast division maintains the stability of the chloroplast number in plant cells. In the process of chloroplast division, chloroplast division proteins form a ring-like complex in the middle of chloroplasts (Osteryoung and Nunnari, 2003; Chen et al., 2018). Then the ring-like complex constricts, splitting one chloroplast into two daughter chloroplasts (Osteryoung and Pyke, 2014). FtsZ is an essential chloroplast division protein conserved in plants and bacteria (Osteryoung and Nunnari, 2003; Stokes and Osteryoung, 2003). There are two sub-family members in plants, including FtsZ1 and FtsZ2 (Osteryoung and McAndrew, 2001; Stokes and Osteryoung, 2003). The correct localization is essential for chloroplast division proteins to function *in vivo* (Zhang et al., 2013, 2016; Chen et al., 2019).

Immunofluorescence staining is a widely used method to study the localization of proteins in the cell. It is frequently used to study the localization of chloroplast division proteins (Vitha et al., 2001; Li et al., 2016; Wang et al., 2017; Liu et al., 2022). Compared with transforming plants with fluorescent protein tags, immunofluorescence staining does not need to obtain transgenic plants, so it is a good choice to study the localization of proteins in plants that cannot be transformed or are untransformed. Moreover, fluorescent protein tags may change the structure and function of the fused protein (Hanson and Ziegler, 2004), and interfere their localization in the cell. However, immunofluorescence staining can reflect the natural and real localization of proteins *in vivo*.

Because of the cell wall of plant cells, it is not easy for the antibodies to enter the cell and recognize the target proteins. Immunofluorescence staining method with wax-embedded tissue sections was used to observe the localization of the chloroplast division proteins FtsZ in plants (Vitha et al., 2001). However, this kind of method takes a long time, they need several days, and the operation is cumbersome and laborious (Ovecka et al., 2014).

In comparison with the tissue sectioning method, a new method using isolated protoplasts for the immunofluorescence staining of chloroplast proteins was developed (Li et al., 2016). This method was used in studying the localization of chloroplast division proteins (Wang et al., 2017; Liu et al., 2022). Compared with the immunofluorescence staining method with wax-embedded tissue sections, this method with enzymatic digestion of cell wall is much simpler and less time-consuming. The results can be obtained within only 1 day. However, due to the special composition and structure of plant cell walls, protoplasts of some plants, especially the woody plants, are hard to obtain. Therefore, this method has some limitations.

To overcome the influence of plant cell walls, we developed another immunofluorescence staining method by directly breaking the leaf tissue into irregular small pieces for immunofluorescence staining. There is no need of embedding the tissue in wax and sectioning, and preparation of poly-L-lysine coated slides and protoplasts by enzyme digestion. The entire experiment can be completed in about 8 h. In addition, the difficulty of operation is greatly reduced. The demand for experimental materials is very low, and a leaf area of 1–2 cm^2^ is enough. We can observe the localization of FtsZ1 in many species, including poplar, elm, *Broussonetia papyrifera* and *Ginkgo biloba* with this method. Thus, this new method should have a wide range of applications.

## Materials and Methods

### Plant Materials and Growth Conditions

*Arabidopsis thaliana* plants used are Columbia-0 (Col) wild type, *pdv2-3* and *arc3-1* (Pyke and Leech, 1994; Chang et al., 2017). *Arabidopsis thaliana*, *Nicotiana benthamiana*, *Brassica oleracea* var. *capitata* L., poplar 84K *(Populus alba* × *P. tremula* var. *glandulosa)* were grown in soil at 22°C with 40–60% relative humidity and 16-h-light/8-h-dark cycles. *Allium tuberosum* Rottler ex Spreng., *Allium sativum* L., *Lactuca sativa* var. *longifoliaf* Lam, *Apium graveolens* L., *Brassica juncea* (L.) Czern., *Brassica juncea* (L.) Czern., *Brassica rapa* var. *oleifera* DC. and *Spinacia oleracea* L. were bought from a vegetable shop. *Dianthus caryophyllus* L. were bought from a flower shop. All other plants were grown in a greenhouse.

### Leaf Breaking and Fixation

All the steps were carried out at room temperature. Leaf tissues were harvested and immediately immersed in fixation solution [0.4 M Mannitol, 20 mM KCl, 20 mM MES (pH 5.7), 4% paraformaldehyde] in petri dishes and cut into pieces by blades. The sharp blades were surgical blades (K3-23, Shanghai Pudong Jinhuan Medical Products Company Limited). The saw-shaped blades were taken from GLAD ClingWrap (W300N, Clorox China Limited). The broken tissue and the fixation solution were transferred together into a 1.5 mL tube and kept in the dark for 1 h.

### Antibody Incubation

Leaf tissues were gently washed with 1 mL 1 × PBS (137 mM NaCl, 2.7 mM KCl, 10 mM Na_2_HPO_4_•12H_2_O, 2 mM KH_2_PO_4_, pH = 7.4) three times and 5 min each time at least. The sample was covered with 1 mL blocking solution (5% BSA in 1 × PBS with 0.15% Triton X-100) and incubated at room temperature for 30 min. The samples were then incubated with 200 μL anti-FtsZ1 antibodies (Liu et al., 2022) (a dilution of 1:100 with blocking solution) for 2 h in darkness at room temperature. Next, samples were washed with 1 × PBS or 5% BSA in 1 × PBS three times. Then the samples were incubated with 100 μL goat anti-rabbit FITC-conjugated secondary antibodies (JX3004, Jiaxuan Biotech, Beijing) (1:100 dilution in 5% BSA) for 1 h in the dark. The samples were washed with 1 × PBS or 5% BSA in 1 × PBS three times.

### Tissue Lysis

In total, 1 mL EDTA•Na_2_ (pH 9.0) was added to samples and incubated at 55°C for 1 h to break the middle lamellar between the cells and lyse the tissue. After removing the EDTA•Na_2_ solution, 1 mL fresh home-made anti-fade mounting medium (5 mM Na-Ascorbate, 15 mM Na_2_HPO4 pH 9.0, 50% glycerin) was added to the tube. Samples were stored at 4°C. Images were taken with a microscope (NEXCOPE NE910, Olympus) equipped with a 40× objective and an E3ISPM camera. Image analysis was carried out using ImageJ^1^ (version 1.52V) and Photoshop (Adobe Photoshop CC 2015).

## Results

### The Procedures of Immunofluorescence Staining

**FIGURE 1:**
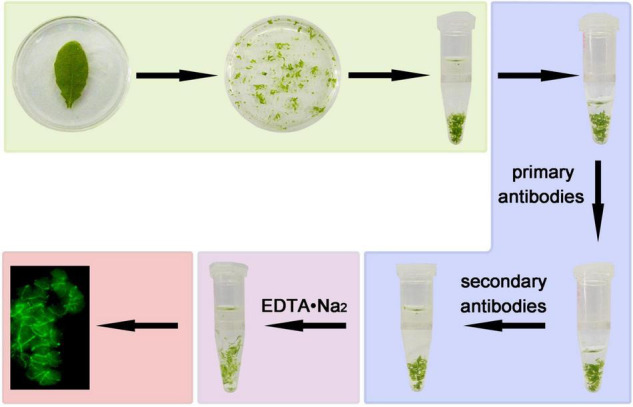
A diagram of the steps of immunofluorescence staining. Leaf in fixation solution were broken by serrated blade, then transferred to a tube and kept for 1 h. The fixed tissues were blocked for 30 min, incubated with anti-FtsZ1 antibodies and FITC-labeled secondary antibodies. The leaf tissues were then decomposed by EDTA•Na_2_ (pH 9.0). Signals of immunofluorescence staining were observed with a microscope.

**FIGURE 2:**
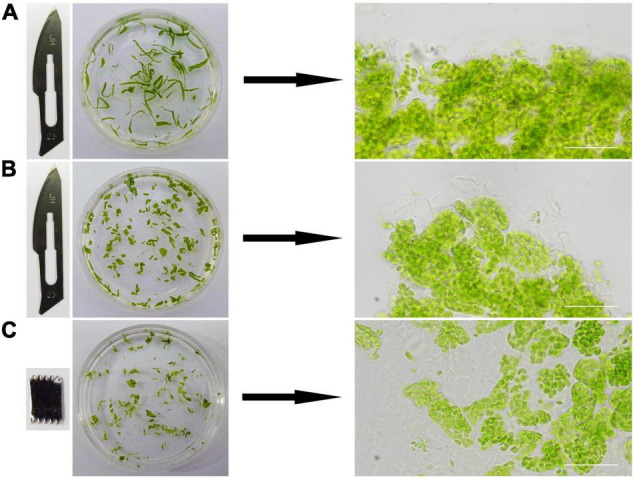
The effects of different cutting methods. Different blades and cutting methods were tested to cut leaves into different shapes. The leaves were cut into filaments **(A)** and squares **(B)** by a sharp blade, and irregular pieces **(C)** by a saw. Images on the right are microscopic views. Bars = 50 μm.

We developed an improved method of immunofluorescence staining directly with leaf tissue. This method does not need wax-embedded tissue sections, separation of protoplasts and preparation of poly-L-lysine slides in advance. The leaf tissue is directly chopped with a serrated blade in fixation solution and fixed instantly, and incubated with primary antibodies and FITC-labeled secondary antibodies. Finally, the intercellular pectin between cells is decomposed by EDTA•Na_2_ and heated to dissociate the cells for a better view of the signals in the cell (Pyke and Leech, 1991; Tsugama et al., 2011; Gao et al., 2013; Figures 1, 2). The method is very simple and only requires a small amount of material.

### It Is Better to Cut the Leaf Tissue With a Saw-Shaped Blade

We tried different methods to cut the leaf tissues. At first, a sharp blade was used and the leaf tissues were cut into narrow pieces or small squares (Figures 2A,B). A saw-shaped blade was also used for the cutting and the leaves were cut into irregular small pieces (Figure 2C). The edges of the leaf tissues cut by a sharp blade were regular and sharp (Figures 2A,B). In contrast, those cut by a saw-shaped blade were irregular and coarse (Figure 2C). So, its apparent that the leaf tissues cut by a saw-shaped blade had much more surface area and cells directly exposed to the solutions and the reagents, which could lead to a better result in the end. This had been confirmed by the final experimental results.

This step required only a few minutes. For tender leaves like Arabidopsis leaves, it can be finished in 1 min. For the hard leaves of woody plants, such as the leaves of poplar, it can be finished in 3–5 mins.

### Tissue Lysis Facilities the Observation of the Signal

**FIGURE 3:**
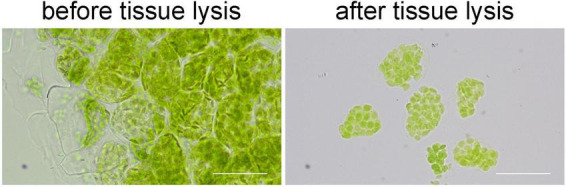
The effects of tissue lysis. After the lysis, cells can be separated easily for a clear view. Bars = 50 μm.

**FIGURE 4:**
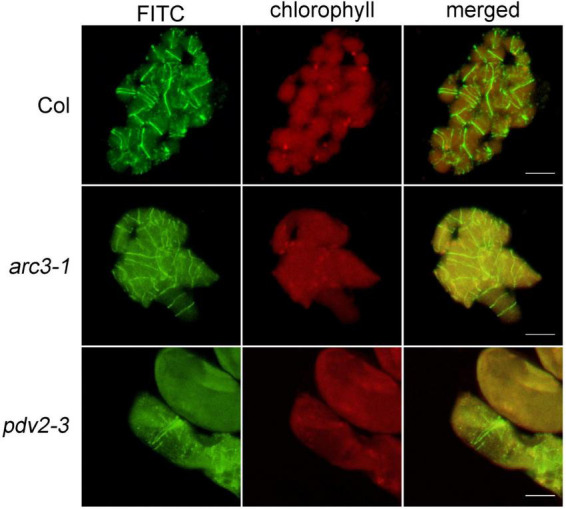
Immunofluorescence staining of FtsZ1 in the wild type, *arc3-1* and *pdv2-3* in Arabidopsis. Bars = 10 μm.

**FIGURE 5:**
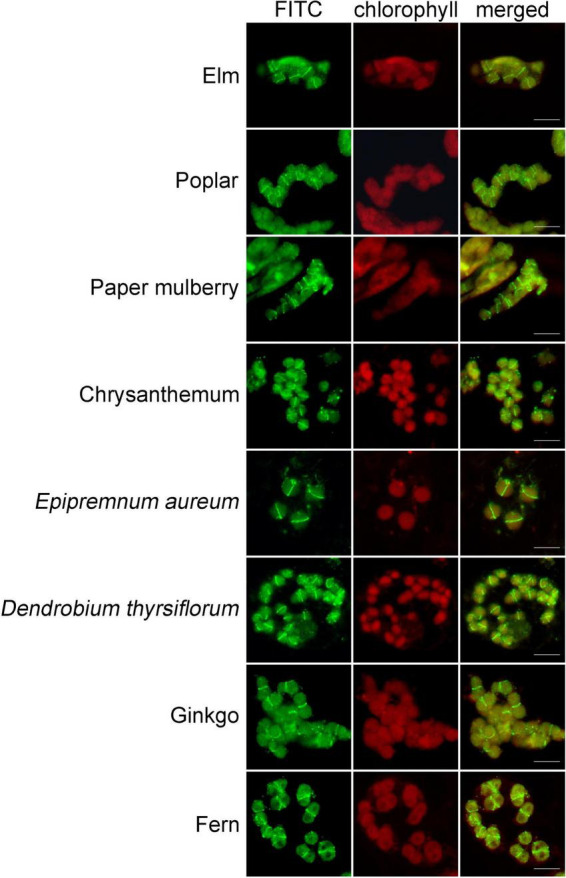
Immunofluorescence staining of FtsZ1 in various plant species. These species are *Ulmus pumila* L., *Populus alba* × *P. tremula* var. *glandulosa*, *Broussonetia papyrifera* (L.) L’Hér. ex Vent., *Dendranthema morifolium* (Ramat.) Tzvel., *Epipremnum aureum* (Linden et Andre) Bunting, *Dendrobium thyrsiflorum* Rchb. f., *Ginkgo biloba* L., *Pteridium aquilinum* (L.) Kuhn var. *latiusculum* (Desv.) Underw. ex Heller. Bars = 10 μm.

When we directly observed the samples after immunofluorescence staining, we found that it was not easy to see the signal of immunofluorescence staining clearly. Because the cells were sticked together in the leaf tissue, the background signals were high and the real signals were blurry. Therefore, we treated the stained tissue with EDTA•Na_2_ (pH 9.0) at 55°C for 1 h. This treatment broke the pectin in the middle lamellar, so the cells can be separated easily (Figure 3). To our surprise, the signal of immunofluorescence staining was not affected by this treatment (Figures 4, 5), and it looked much clear since the cells were dispersed.

### Immunofluorescence Staining of FtsZ1 in Arabidopsis by the Newly Developed Method

In order to confirm the feasibility of this method, Arabidopsis wild type was used at first to observe the localization of the chloroplast division protein FtsZ1. The results indicated that FtsZ1 protein formed a ring and localized to the middle of chloroplasts (Figure 4), as previous reported with other immunofluorescence staining methods (Vitha et al., 2001; Li et al., 2016). Next, we tested this new method with Arabidopsis chloroplast division mutants *arc3* and *pdv2* (Pyke and Leech, 1994; Chang et al., 2017). In these mutants, chloroplasts are increased in size and decreased in number, and the FtsZ rings are irregular (Miyagishima et al., 2006; Zhang et al., 2013; Wang et al., 2017). The results showed that FtsZ1 was distributed in large chloroplasts in the *arc3-1* mutant as multiple parallel rings (Figure 4). In the *pdv2-3* mutant, the rings of FtsZ1 were distributed in the middle of the large chloroplasts (Figure 4). The localization results of FtsZ1 in *arc3-1* and *pdv2-3* obtained by this method were also similar to those of previously reported (Miyagishima et al., 2006; Zhang et al., 2013). Therefore, the new immunofluorescence staining method developed in this study is suitable for observing the localization of chloroplast division proteins in Arabidopsis.

### The New Immunofluorescence Staining Method Can Be Widely Used in Many Other Species

To learn whether this newly-developed method can be applied in other plants, we used the leaves from a wide variety of many other plant species, including some woody plants, for the test. Since FtsZ1 is a well conserved protein in plants, we used the FtsZ1 antibodies as the primary antibodies. In many of the plants, a good signal of FtsZ1 were clearly seen in the cells (Figure 5). FtsZ1 formed a ring in middle of the chloroplasts of those plants, which is consistent with the localization of FtsZ1 in Arabidopsis. Figure 5 shows the immunofluorescence staining of some selected plants. Table 1 shows a partial list of the tested plants which showed a good signal of FtsZ1. As we can see, those plants include moss, fern, gymnosperm and angiosperms. For some woody plants, such as poplar and gingko, a good signal could also be obtained (Figure 5). Therefore, this newly-developed method can be applied to many plant species and it has a wide applicability.

**TABLE 1:** A partial list of the species in which FtsZ1 can be detected with good signals by the newly-developed immunofluorescence staining method.

A partial list of the plant species
*Pisum sativum* L.
*Glycine max* (L.) Merr.
*Nerium oleander* L.
*Gossypium hirsutum* L.
*Dendranthema morifolium* (Ramat.) Tzvel.
*Lactuca sativa* var. *longifoliaf* Lam
*Ailanthus giraldii* Dode
*Prunus davidiana* (Carrière.) Franch.
*Prunus persica*
*Nicotiana benthamiana*
*Broussonetia papyrifera* (L.) L’Hér. ex Vent.
*Apium graveolens* L.
*Cardamine hirsuta* L.
*Brassica oleracea* var. *capitata* L.
*Brassica juncea* (L.) Czern.
*Brassica rapa* var. *oleifera* DC.
*Dianthus caryophyllus* L.
*Spinacia oleracea* L.
*Boehmeria nivea* (L.) Gaudich.
*Salix matsudana* var. *pseudomatsudana* (Y. L. Chou & Skvortzov) Y. L. Chou
*Populus alba* × *Populus tremula* var. *glandulosa*
*Ulmus pumila* L.
*Murraya exotica* L.
*Citrus reticulata* Blanco
*Zanthoxylum bungeanum* Maxim.
*Cinnamomum camphora* (L.) J. Presl
*Oxalis corymbosa* DC.
*Phalaenopsis aphrodite* Rchb. f.
*Dendrobium thyrsiflorum* Rchb. f.
*Alstroemeria aurea* Graham
*Allium tuberosum* Rottler ex Spreng.
*Allium sativum* L.
*Allium schoenoprasum* L.
*Hippeastrum rutilum* (Ker-Gawl.) Herb.
*Chlorophytum comosum* (Thunb.) Baker
*Ophiopogon japonicus* (L. f.) Ker Gawl.
*Cordyline fruticosa* (L.) A. Chev.
*Philodendron selloum* K. Koch
*Epipremnum aureum* (Linden et Andre) Bunting
*Tradescantia fluminensis*
*Livistona chinensis* (Jacq.) R. Br. ex Mart.
*Chamaedorea elegans* Mart.
*Ginkgo biloba* L.
*Pteridium aquilinum* (L.) Kuhn var. *latiusculum* (Desv.) Underw. ex Heller
*Marchantia polymorpha* L.

## Discussion

Immunofluorescence staining is a method widely used to study the localization of proteins in the cells. In this study, we developed a new method for the immunofluorescence staining of chloroplast proteins in plant cells. We found that use a serrated blade, instead of a sharp blade, to cut the leaves into small irregular pieces make the tissues more accessible to the reagents. After the fixation and immunofluorescence staining with the primary and secondary antibodies, those small leaf tissues were lysed by a simple way, which made them easily separated into single cells. This greatly reduced the background noise signal and presented a clear view of the true signal in the cell. Although these treatments seemed to be not new, but they were not used in immunofluorescence staining previously. The combination of these steps made this immunofluorescence staining method superior to the previous ones.

We first tried this method in Arabidopsis to detect the localization of a chloroplast division protein, FtsZ1, in the wild type and two chloroplast division mutants. The localization results are very similar to those obtained by other methods (McAndrew et al., 2001; Vitha et al., 2001; Zhang et al., 2013; Li et al., 2016; Chen et al., 2019). The leaves of Arabidopsis are tender and easy to operate. We then further test the applicability of this method in various many other plants. Good signals were observed in many of those plants, including some woody plants, such as gingko and poplar.

Our new method has some advantages to the previous methods. Firstly, it is very easy and simple. Although the protoplast-based immunofluorescence staining method is easy too, our method doesn’t require preparation of protoplasts and poly-lysine coated glass slides. It is much easier than the wax-embedding and sectioning method too. Secondly, it is very fast. The whole process can be finished in several hours. Thirdly, it only needs a small piece of leaf tissue. An area of 1–2 cm^2^ will be enough for the experiment. Fourthly, it doesn’t need enzymes to lyse the leaf tissues. The tissues are directly broken by a saw-shaped blade into small pieces and then simply lysed by EDTA•Na_2_ and heating. So, it is very cost-effective. Fifthly, due to the existence of the cell wall, protoplasts are difficult to prepare in many plants, such as woody plants and liverworts. Our tissue-breaking and tissue lysis method can overcome this problem to a large extent and it can be used in a wide variety of plants. Theoretically, this method can also be used for the localization study of many other proteins in the cell. It provides an additional and good choice of the methods for the study of protein localization.

## Data Availability Statement

The raw data supporting the conclusions of this article will be made available by the authors, without undue reservation.

## Author Contributions

HG designed the experiments. LW, MT, WH, and JA carried out the experiments. LW, XL, and HG prepared the manuscript. All authors contributed to the article and approved the submitted version.

## Conflict of Interest

The authors declare that the research was conducted in the absence of any commercial or financial relationships that could be construed as a potential conflict of interest.

## Publisher’s Note

All claims expressed in this article are solely those of the authors and do not necessarily represent those of their affiliated organizations, or those of the publisher, the editors and the reviewers. Any product that may be evaluated in this article, or claim that may be made by its manufacturer, is not guaranteed or endorsed by the publisher.

## Footnotes

1
http://rsbweb.nih.gov/ij/
